# Diagnostic value of magnetic resonance parametric mapping for non-invasive assessment of liver fibrosis in patients with primary sclerosing cholangitis

**DOI:** 10.1186/s12880-021-00598-0

**Published:** 2021-04-07

**Authors:** Narine Mesropyan, Patrick Kupczyk, Guido M. Kukuk, Leona Dold, Tobias Weismueller, Christoph Endler, Alexander Isaak, Anton Faron, Alois M. Sprinkart, Claus C. Pieper, Daniel Kuetting, Christian P. Strassburg, Ulrike I. Attenberger, Julian A. Luetkens

**Affiliations:** 1 Department of Diagnostic and Interventional Radiology, University Hospital Bonn, Venusberg-Campus 1, 53127 Bonn, Germany; 2 Department of Radiology, Kantonsspital Graubünden, Chur, Switzerland; 3 Department of Internal Medicine I, University Hospital Bonn, Venusberg-Campus 1, 53127 Bonn, Germany

**Keywords:** Primary sclerosing cholangitis, Magnetic resonance elastography, Extracellular volume fraction

## Abstract

**Background:**

Primary sclerosing cholangitis (PSC) is a chronic cholestatic liver disease, characterized by bile duct inflammation and destruction, leading to biliary fibrosis and cirrhosis. The purpose of this study was to investigate the utility of T1 and T2 mapping parameters, including extracellular volume fraction (ECV) for non-invasive assessment of fibrosis severity in patients with PSC.

**Methods:**

In this prospective study, patients with PSC diagnosis were consecutively enrolled from January 2019 to July 2020 and underwent liver MRI. Besides morphological sequences, MR elastography (MRE), and T1 and T2 mapping were performed. ECV was calculated from T1 relaxation times. The presence of significant fibrosis (≥ F2) was defined as MRE-derived liver stiffness ≥ 3.66 kPa and used as the reference standard, against which the diagnostic performance of MRI mapping parameters was tested. Student *t* test, ROC analysis and Pearson correlation were used for statistical analysis.

**Results:**

32 patients with PSC (age range 19–77 years) were analyzed. Both, hepatic native T1 (r = 0.66; *P* < 0.001) and ECV (r = 0.69; *P* < 0.001) correlated with MRE-derived liver stiffness. To diagnose significant fibrosis (≥ F2), ECV revealed a sensitivity of 84.2% (95% confidence interval (CI) 62.4–94.5%) and a specificity of 84.6% (CI 57.8–95.7%); hepatic native T1 revealed a sensitivity of 52.6% (CI 31.7–72.7%) and a specificity of 100.0% (CI 77.2–100.0%). Hepatic ECV (area under the curve (AUC) 0.858) and native T1 (AUC 0.711) had an equal or higher diagnostic performance for the assessment of significant fibrosis compared to serologic fibrosis scores (APRI (AUC 0.787), FIB-4 (AUC 0.588), AAR (0.570)).

**Conclusions:**

Hepatic T1 and ECV can diagnose significant fibrosis in patients with PSC. Quantitative mapping has the potential to be a new non-invasive biomarker for liver fibrosis assessment and quantification in PSC patients.

## Background

Primary sclerosing cholangitis (PSC) is a rare cholestatic liver disease, leading to biliary fibrosis and cirrhosis. PSC is believed to be immune-mediated, however, the etiopathogenesis of the disease has still not been completely investigated and remains unclear. The main feature of PSC is a long-term, progressive inflammation followed by fibrosis of the intra- and extrahepatic bile ducts [1, 2]*.* PSC has a strong male predominance and is often associated with other immune-mediated diseases such as inflammatory bowel disease (IBD, e.g. ulcerative colitis) and autoimmune hepatitis (AIH). To date, there are several reports of therapy showing effect in PSC, but no established medical therapy with proven effect on transplant-free survival [3, 4]. According to the guidelines of the European (EASL) and American (AASLD) Associations for the Study of Liver Diseases, magnetic resonance imaging (MRI) including MR-cholangiography has been established as the standard imaging modality when PSC is suspected [5, 6]. As for any other chronic liver disease, early detection of fibrotic changes of liver parenchyma with fibrosis staging, evaluation of disease activity and severity, prognosis estimation as well as malignancy exclusion (e.g. cholangiocarcinoma and/or hepatocellular carcinoma) are of great clinical importance. Therefore, diagnostic approaches enabling these efficiently and non-invasively in the same clinical setting without adding costs and burdens in patients’ care are required [7, 8].

Over the last decades, MRI techniques have undergone significant advancement from a qualitative to quantitative approach, offering the opportunity for the development of objective and reproducible imaging biomarkers that can be incorporated into clinical routine [9]. To date, magnetic resonance elastography (MRE) is considered to be a safe noninvasive technique with excellent diagnostic accuracy for liver fibrosis assessment [10–13], and routine liver biopsy is no longer recommended for fibrosis staging in PSC [5, 6]. However, MRE requires additional expensive equipment and is only available in specialized centers. Therefore, ubiquitously available quantitative imaging techniques might be desirable that can encompass a major portion of the liver.

Initially extensively used in cardiac imaging for the detection and quantification of cardiac fibrosis and inflammation, quantitative T1 and T2 mapping techniques [14], might also be promising MRI techniques for the evaluation of liver parenchyma. According to current studies, hepatic fibrosis increases the T1 and T2 relaxation time of liver parenchyma due to an increase of extracellular matrix and protein concentration [15–17]. T1 mapping techniques also allow the estimation of extracellular volume fraction (ECV) from native and post-contrast T1. ECV is a biomarker of the extracellular space and reflects tissue volume which is not taken by cells [18]. ECV can be calculated from the change in relaxation rate (R1 = 1/T1) of blood and parenchyma corrected for the hematocrit [17, 19]. Although several studies investigated the role of T1 and T2 mapping techniques as well as ECV for liver fibrosis assessment [16, 17, 20–23], reliable data investigating these techniques in patients with PSC are still missing. Therefore, the goal of the present study was to explore the diagnostic value of MRI mapping parameters, including ECV to diagnose significant fibrosis in PSC patients using MRE-derived liver stiffness as a reference standard.

## Methods

This study was approved by the institutional review board and was conducted in compliance with the ethical standards set in the 1964 Declaration of Helsinki as well as its later amendments. Written informed consent was obtained from all participants prior to MRI examination. Consecutive patients of the University Hospital of Bonn with diagnosis of PSC were prospectively enrolled from January 2019 to July 2020. Diagnosis of PSC was based on diagnostic criteria of PSC established by the EASL [6]. Patients with additional features of AIH and accompanying IBD were also included. Exclusion criteria were as follows: (1) concomitant diagnosis of other chronic liver diseases, including hepatic steatosis and iron overload; (2) contraindications for MRI; (3) acute ascending cholangitis; (4) cholangiocarcinoma or hepatocellular carcinoma; (5) previous liver transplant; (6) small-duct PSC; (7) insufficient imaging quality or absence of laboratory tests at the time of MRI examination. Additionally, data of liver stiffness measurements derived by transient elastography (TE, FibroScan) were analyzed. A cutoff value of 8.6 kPa was chosen to differentiate between patients without (< F2) and with (≥ F2) significant fibrosis [24]. Biochemical blood analyses were performed using standard tests and non-invasive serologic fibrosis scores (aspartate aminotransferase to platelet ratio index (APRI), fibrosis index based on the 4 factor (FIB-4) and aspartate aminotransferase and alanine aminotransferase ratio (AST/ALT ratio (de-Ritis)) were calculated [25–27]. All clinical data and laboratory markers were recorded from the patient charts. None of the patients of the study cohort had acute exacerbation of PSC, IBD and AIH at the time of MRI examination based on clinical and laboratory findings and received symptomatic therapy according to current guidelines [6].

### Multiparemetric MRI

All liver MRI were performed on a clinical whole-body 1.5 T system (Ingenia, Philips Healthcare) equipped with 32-channel abdominal coil with digital interface for signal reception. Liver MRE and T1 and T2 mapping were performed in addition to morphological sequences. For liver MRE, a 2D gradient-recalled echo with added cyclic motion encoding gradients (MEGs) sequence with the following parameters was applied: time of repetition (TR)/time of echo (TE) 50/20 ms, flip angle (FA) 20°, parallel imaging factor 2.3, active driver frequency 60 Hz, active driver power 100%, field of view (FOV) 450 × 403 × 32 mm, acquired voxel size 1.50 × 4.74 × 10 mm, reconstructed voxel size 1.17 × 1.17 × 10 mm^3^, scan duration/breath hold 15.3 s, 3 slices. The system configuration was based on an active pneumatic driver connected via plastic tube with a passive driver, which was placed at the patient's right upper quadrant. MRE involves (a) generation of shear waves in the tissue, (b) acquisition of MR images, (c) depicting the propagation of the induced shear waves, and (d) postprocessing of the share waves to generate quantitative liver stiffness maps using implemented vendor´s software (MR elastography View, Philips Healthcare). For hepatic T1 mapping, we used a heart rate independent 10-(2)-7-(2)-5-(2)-3-(2) modified Look-Locker inversion recovery (MOLLI) acquisition scheme with internal triggering [28]. Technical parameters were as follows: TR/TE 1.92/0.84 ms, FA 20°, parallel imaging factor 2, acquired voxel size 1.98 × 2.45 × 10 mm^3^, reconstructed voxel size 1.13 × 1.13 × 10 mm^3^, scan duration/breath hold 14 s. For the post-contrast T1 maps the same technique was used after 10 min of contrast agent application in the same positions as pre-contrast examinations. For contrast enhanced T1 mapping, a gadolinium-based contrast agent (Gadobutrol, 1.0 mmol/ml solution with 0.1 mmol per kilogram of body weight, Gadovist, Bayer Healthcare Pharmaceuticals) was administered as a single bolus with an injection rate of 1.5 ml/s. Hepatic T2 mapping was performed using a six-echo gradient spin echo sequence (GraSE) with the following parameters [29]: TR/TE 450/16 ms, inter-echo spacing 16 ms, FA 90°, parallel imaging factor 2.5, acquired voxel size 1.98 × 2.01 × 10 mm, reconstructed voxel size 0.88 × 0.88 × 10 mm, scan duration/breath hold 15/3 × 5 s. Hepatic quantitative maps were acquired in a single transversal slice slightly above the liver hilum. Relaxation maps were reconstructed at the scanner console.

### Image analysis

An experienced board-certified radiologist (J.A.L, 8 years of experience in abdominal MRI) performed image analyses, blinded to the clinical data. For the assessment of T1 and T2 relaxation times, the mean relaxation time of three representative regions of interest (ROI) was calculated. ROIs were drawn centrally in the hepatic segments II, IVa and VII within liver parenchyma away from confounding factors like organ borders, vessels or bile ducts. Blood pool T1 values were derived from the abdominal aorta. ECV was calculated with ROI-based on pre- and post-contrast T1 values according to the previously published equation [30]. Hematocrit samples were derived on the same day prior to MRI examination. Liver tissue stiffness values were derived from stiffness confidence map by drawing largest possible ROIs (≥ 1 cm^2^) in at least three different representative regions of the liver. Based on MRE-derived liver stiffness, all study participants were divided into two groups, first, without (< fibrosis stage F2) and second, with significant fibrosis (≥ F2). To differentiate between patients with and without significant liver fibrosis a cutoff value of 3.66 kPa was chosen [31].

### Statistical analysis

All data were analyzed using software (SPSS Statistics, version 25, IBM, MedCalc, version 19.1.3, MedCalc). Patient characteristics are presented as mean ± standard deviation, as absolute frequency or median, as appropriate. Student *t* test was used for comparison of continuous variables between two different groups. Dichotomous variables were compared using the χ^2^ test (with the cell count > 5) and Fisher test (with a cell count ≤ 5). Bivariate Pearson correlation coefficient or Spearman’s rank correlation coefficient were used for a correlation analyses, as appropriate. Receiver operating characteristic analysis (ROC) was used to determine the cut-points with the highest combined sensitivity and specificity, positive predictive values (PPV), negative predictive values (NPV) and accuracy using MRE-derived liver stiffness as a reference standard. DeLong method was used to compare areas under the curves (AUCs) [32]. MRE-derived liver stiffness as well as liver stiffness derived by TE were the reference standards against which the diagnostic performance of MRI-derived mapping parameters of liver was tested. The level of statistical significance was set to *P* < 0.05.

## Results

### Cohort characteristics

A total of 32 patients with diagnosis of large-duct PSC were included in this study. Based on MRE-derived stiffness values, 40.6% (13/32) patients had no (< F2) and 59.4% (19/32) had significant (≥ F2) fibrosis. 15.8% (3/19), 21.1% (4/19), and 63.1% (12/19) patients had fibrosis stages F2, F3 and F4, respectively. 18.7% (6/32) patients had additional features of AIH. There were 61.5% (8/13) patients with intrahepatic biliary changes only and 38.5% (5/13) patients with both intra- and extrahepatic bile duct changes in patients without significant fibrosis (< F2) and 73.7% (14/19) and 26.3% (5/19) in the group of patients with significant fibrosis (≥ F2), respectively. In patients without significant fibrosis (< F2), there were 15.4% (2/13) patients with and 84.6% (11/13) with no imaging features of portal hypertension (varices, splenomegaly, and/ or ascites). In patients with significant fibrosis (≥ F2) there were 52.6% (10/19) patients with and 47.4% (9/19) patients without imaging features of portal hypertension (*P* = 0.03). The mean age of the disease onset in the group of patients without significant fibrosis was 38.4 ± 7.5 years, with significant fibrosis 31.1 ± 12.5 years. At the time of MRI examination, 40.6% (13/32) patients received therapy with ursodeoxycholic acid (UDCA) alone; 31.3% (10/32) patients received a combination of 5-aminosalicylic acid (5-ASA) with UDCA due to accompanying IBD; 12.5% (4/32) patient the combination of corticosteroids (budesonide) with UDCA due to overlap with AIH. 20.0% (5/32) patients received no therapy at the time of MRI examination. Clinical characteristics are summarized in Table1. Additionally, 18/32 patients had TE examination within 6 month to MRI examination (the mean interval between MRI examination and TE was 66.3 ± 48.0 days).

**Table 1:** Clinical characteristics of patients without significant fibrosis (< F2) and with significant fibrosis (≥ F2)

Variable	PSC patients without significant fibrosis (< F2, n = 13)	PSC patients with significant fibrosis (≥ F2, n = 19)	*P* value
Age (years)	43.1 ± 12.8	39.5 ± 17.5	0.531
Body mass index (kg/m^2^)	23.8 ± 2.9	24.8 ± 3.8	0.439
*Sex*			0.246
Male	7 (53.8%)	4/19 (21.1%)	
Female	6 (46.2%)	15 (78.9%)	
Hematocrit level (%)	43 ± 4	42 ± 6	0.526
Bilirubin (mg/dl)	0.56 ± 0.25	1.19 ± 0.98	0.031
ALT (U/l)	52.1 ± 55.3	118.1 ± 92.9	0.029
AST (U/l)	33.4 ± 11.4	81.9 ± 46.6	0.001
GGT (U/l)	155.5 ± 116.7	240.3 ± 180.5	0.147
Platelets cells × 10^9^/l	291.2 ± 81.1	248.5 ± 130.7	
C-reactive protein level (mg/l)	12.4 ± 22.8	2.1 ± 1.5	0.221
AP (U/l)	285.8 ± 181.6	140.8 ± 45.4	0.013
Creatinine (mg/dl)	0.79 ± 0.09	0.79 ± 0.14	0.946
Albumin (g/l)	45.9 ± 3.2	42.8 ± 5.3	
International normalized ratio	1.08 ± 0.28	1.05 ± 0.12	0.683
ASL/ALT (de-Ritis)	0.85 ± 0.28	0.82 ± 0.33	0.798
FIB-4	0.85 ± 0.62	1.84 ± 2.86	0.232
MELD	6.69 ± 2.21	7.47 ± 2.46	0.919
APRI	0.31 ± 0.19	0.94 ± 1.10	0.052
Mayo score	− 1.09 ± 0.54	0.03 ± 1.34	0.012

Continuous data are means ± standard deviations. Nominal data are absolute frequencies with percentages in parentheses

MELD, Score Model of End Stage Liver Disease; ALT, Alanine aminotransferase; AST, Aspartate aminotransferase; AP, Alkaline phosphatase, GGT, Gamma-glutamyltransferase; APRI, aspartate aminotransferase to platelet ratio index; FIB-4, Fibrosis-4-Score; ASL/ALT (de-Ritis), De-Ritis ratio

### Transient elastography results

Based on liver stiffness measurements derived by TE 44.4% (8/18) patients had no (< F2) and 55.6% (10/18) had significant fibrosis (≥ F2). The mean value of liver stiffness measurements derived by TE in patients without (< F2) was 5.7 ± 0.8 kPa and in patients with significant fibrosis (≥ F2) 23.1 ± 20.3 kPa (*P* = 0.024). We found significant correlations between liver stiffness measurements derived by TE and MRE (r = 0.78, *P* < 0.001) as well as hepatic ECV (r = 0.52, *P* = 0.026). ECV was significant higher in patients with significant fibrosis according to TE (32.2 ± 5.7% vs. 27.1 ± 1.4%; *P* = 0.023).

Based on TE analysis, hepatic ECV revealed a diagnostic performance with an AUC of 0.815, a sensitivity of 77.8% and a specificity of 66.7% to diagnose significant fibrosis (cutoff value: 27.7%). Hepatic native T1 showed also high diagnostic performance with an AUC of 0.870, a sensitivity of 77.8% and a specificity of 88.9% to diagnose significant fibrosis (cutoff value: > 559 ms). Hepatic T2 achieved an AUC of 0.753, a sensitivity of 55.6% and a specificity of 100.0% (cutoff value: > 53.3 ms).

### MRI results

**Fig. 1:**
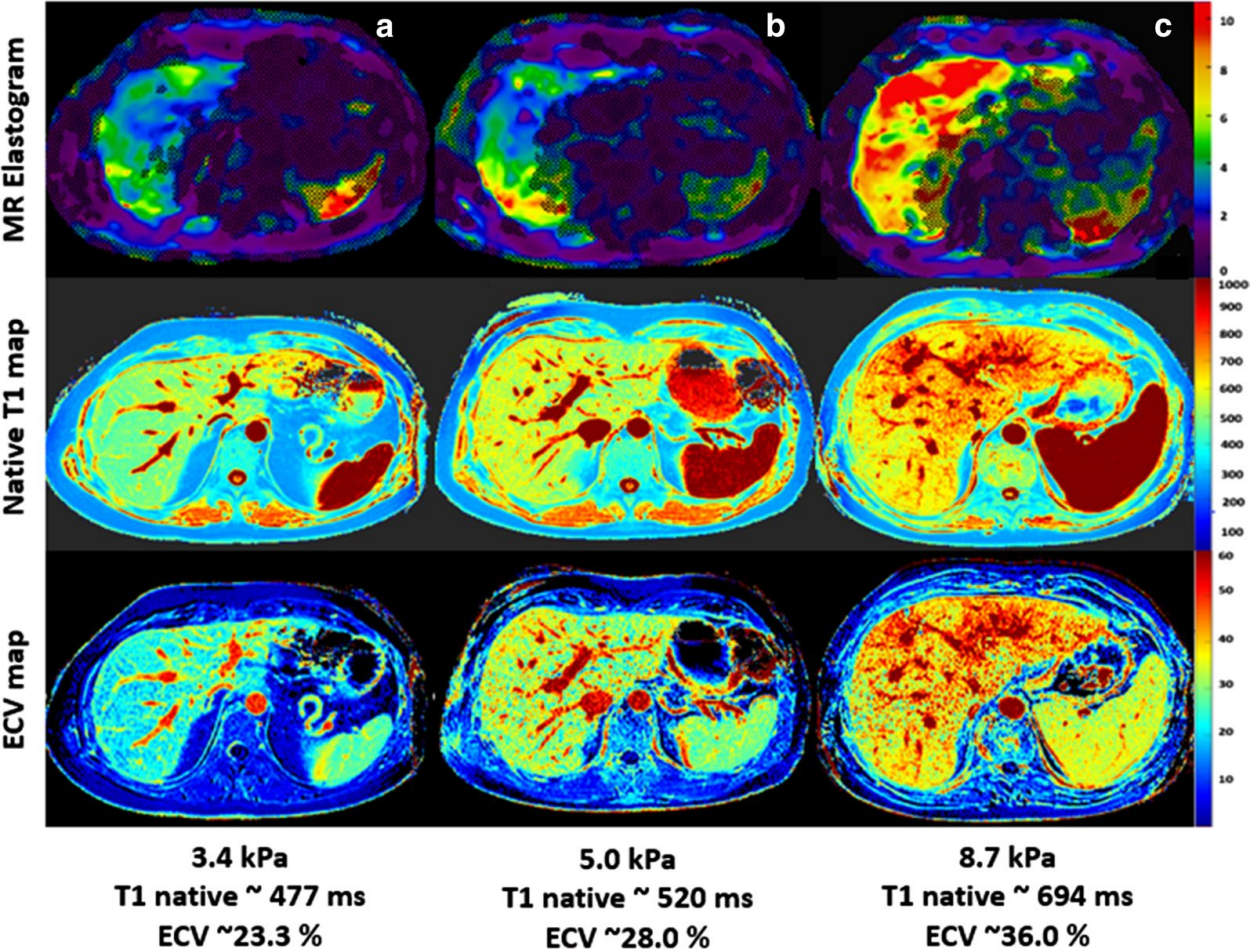
Representative images of hepatic native T1 and extracellular volume (ECV) maps and magnetic resonance elastogram (MRE) from patient without significant fibrosis (< F2, **a**) and patients with significant fibrosis (≥ F2, **b** and **c**). Figure exemplarily illustrates alterations in quantitative hepatic parameters found in our study. ECV: extracellular volume fraction, F: fibrosis stage

Hepatic native T1 as well as ECV were remarkably increased in the group of patients with significant fibrosis (≥ F2) compared to the group of patients without significant fibrosis (< F2): 559.6 ± 56.3 ms vs. 522.8 ± 33.2 ms, *P* = 0.043, and 30.5 ± 4.4% vs. 26.3 ± 1.9%, *P* = 0.003, respectively (see also Fig. 1). We found no significant differences in hepatic T2 relaxation times between both groups (48.9 ± 3.2 ms vs. 52.8 ± 7.9 ms; *P* = 0.108). Also, fat fraction differed not significantly between both groups (4.6 ± 3.5% vs. 3.1 ± 1.9%, *P* = 0.153). All MRI parameters are given in Table 2. A parameter correlation matrix is given in Table 3.

**Table 2:** Hepatic MRI characteristics of patients without (< F2) and with significant fibrosis (≥ F2)

Variable	PSC patients without significant fibrosis (< F2, n = 13)	PSC patients with significant fibrosis (≥ F2, n = 19)	*P* value
MRE-derived liver stiffness (kPa)	3.2 ± 0.3	5.4 ± 1.4	< 0.001
Hepatic native T1 relaxation time (ms)	522.8 ± 33.2	559.6 ± 56.3	0.043
Hepatic extracellular volume fraction (%)	26.3 ± 1.9	30.5 ± 4.4	0.003
Hepatic T2 relaxation time (ms)	48.9 ± 3.2	52.8 ± 7.9	0.108
Hepatic T2* relaxation time (ms)	30.6 ± 3.3	32.9 ± 8.4	0.370
Proton density fat fraction	4.6 ± 3.5	3.1 ± 1.9	0.153

Continuous data are means ± standard deviations

**Table 3:** Correlation matrix for quantitative MRI parameters and clinical fibrosis scores

Variable	Hepatic native T1		Hepatic T2		Hepatic ECV	
	r value	*P* value	r value	*p* value	r value	*p* value
MRE-derived liver stiffness	0.66	< 0.001	0.41	0.021	0.69	< 0.001
FIB-4	0.21	0.276	0.13	0.501	0.46	0.011
APRI	0.20	0.284	0.18	0.352	0.49	0.005
AST/ALT ratio (de-Ritis)	0.21	0.264	0.33	0.077	0.24	0.199
Mayo score	0.37	0.048	0.41	0.026	0.51	0.004

ECV, extracellular volume fraction. MRE, Magnetic resonance elastography, FIB-4, Fibrosis-4-Score; ASL/ALT ratio (de-Ritis), De-Ritis ratio, APRI, aspartate aminotransferase to platelet ratio index

### Diagnostic performance of MRI-derived mapping parameters

**Fig. 2:**
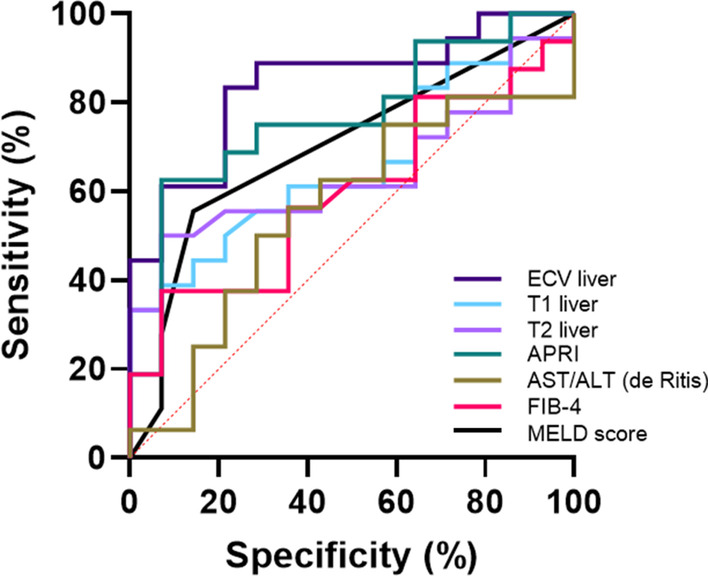
Graphs show receiver operating characteristic curves of different MRI and laboratory markers for diagnosis of significant fibrosis in patients with primary sclerosing cholangitis (≥ F2). Curves are given for hepatic T1 relaxation times (area under curve [AUC]: 0.711), hepatic ECV (AUC: 0.858), hepatic T2 relaxation times (AUC: 0.686), APRI (AUC: 0.787), FIB-4 score (AUC: 0.588), ALT/AST ratio (de-Ritis) (AUC: 0.570), and MELD score (AUC: 0.680). APRI: AST-to-Platelet Ratio Index, FIB-4 score: Fibrosis-4 score, MELD: Model of End Stage Liver Disease

Analysis of the diagnostic performance of MRI-derived mapping parameters for diagnosing significant fibrosis (≥ F2) was performed. According to the ROC analysis among all mapping parameters, hepatic ECV and native T1 demonstrated the best diagnostic performances with an AUC of 0.858 and 0.711, respectively, which were also comparable (*P* = 0.113). Hepatic ECV provided a sensitivity of 84.2% (95% confidence interval (CI) 62.4–94.5%), and a specificity of 84.6% (CI 57.8–95.7%). Hepatic native T1 provided a sensitivity of 52.6% (CI 31.7–72.7%) and specificity of 100.0% (CI 77.2–100.0%). Diagnostic performance of hepatic ECV was significantly higher when compared to the evaluated fibrosis scores FIB-4 and de-Ritis ratio (*P* values: 0.028 and 0.016, respectively) and equal when compared to the APRI and MELD scores (*P* values: 0.523 and 0.123, respectively). Furthermore, in contrast to ECV, diagnostic performance of hepatic native T1 was comparable with that of all evaluated serological fibrosis scores: APRI (0.711 vs. 0.787, *P* = 0.336), FIB-4 (0.711 vs. 0.588, *P* = 0.475), de-Ritis ratio (0.711 vs. 0.570, *P* = 0.370). Hepatic T2 also performed well, however, with diagnostic performance expressed as AUC significantly lower when compared to hepatic ECV (AUC 0.686 vs. 0.858, *P* = 0.006) and equal when compared to hepatic native T1 (0.686 vs. 0.711, *P* = 0.196). Hepatic T2 provided a sensitivity of 57.9% (CI 36.3–76.9%) and a specificity of 92.3% (CI 66.7–98.6%). All values of diagnostic performance statistics for evaluated laboratory and mapping parameters are presented in Table 4, see also Fig. 2.

**Table 4:** Diagnostic performance of different quantitative MRI parameters for and the assessment of liver fibrosis in patients without (< F2) and with significant (≥ F2) fibrosis

Variable	AUC	Cutoff value	Sensitivity (%)	Specificity (%)	PPV (%)	NPV (%)	Accuracy (%)
Hepatic native T1 (ms)	0.711	> 562.7	52.6 (31.7–72.7)	100.0 (77.2–100.0)	100.0 (72.2–100.0)	59.1 (38.7–76.7)	71.9 (54.6–84.4)
Hepatic extracellular volume fraction (%)	0.858	> 27.2	84.2 (62.4–94.5)	84.6 (57.8–95.7)	88.9 (67.2–96.9)	78.6 (52.4–92.4)	84.4 (68.2–93.1)
Hepatic T2 (ms)	0.686	> 52.0	57.9 (36.3–76.9)	92.3 (66.7–98.6)	91.7 (64.6–98.5)	60.0 (38.7–78.1)	71.9 (54.6–84.4)
APRI score	0.787	> 0.41	64.7 (41.3–82.7)	84.6 (57.8–95.7)	84.6 (57.8–95.7)	64.7 (41.3–82.7)	73.3 (55.6–85.8)
FIB-4 score	0.588	> 1.2	35.3 (17.3–58.7)	76.9 (49.7–91.8)	66.7 (35.4–87.9)	47.6 (28.3–67.6)	53.3 (36.1–69.8)
ALT/AST ratio (de-Ritis)	0.570	≤ 0.76	58.8 (36.6–78.4)	69.2 (42.4–87.3)	71.4 (45.4–88.3)	56.3 (33.2–76.9)	63.3 (45.5–78.1)
MELD score	0.680	> 6	52.6 (31.7–72.7)	84.6 (57.8–95.7)	83.3 (55.2–95.3)	55.0 (34.2–74.2)	65.6 (48.3–79.6)

Data in parentheses are 95% confidence interval

PPV, positive predictive value, NPV, negative predictive value, MELD, Score Model of End Stage Liver Disease; APRI, aspartate aminotransferase to platelet ratio index; FIB-4, Fibrosis-4-Score; ASL/ALT ratio (de-Ritis), De-Ritis ratio

## Discussion

The study aimed to investigate the diagnostic value of different MRI mapping parameters including ECV for the evaluation of liver fibrosis using MRE-derived liver stiffness as a reference standard in PSC patients. The main findings of the present study are: (1) hepatic ECV and native T1 correlated strong with MRE-derived liver stiffness and, (2) for the diagnosis of significant fibrosis (≥ F2), hepatic ECV and native T1 revealed the highest diagnostic performance in patients with PSC.

**Fig. 3:**
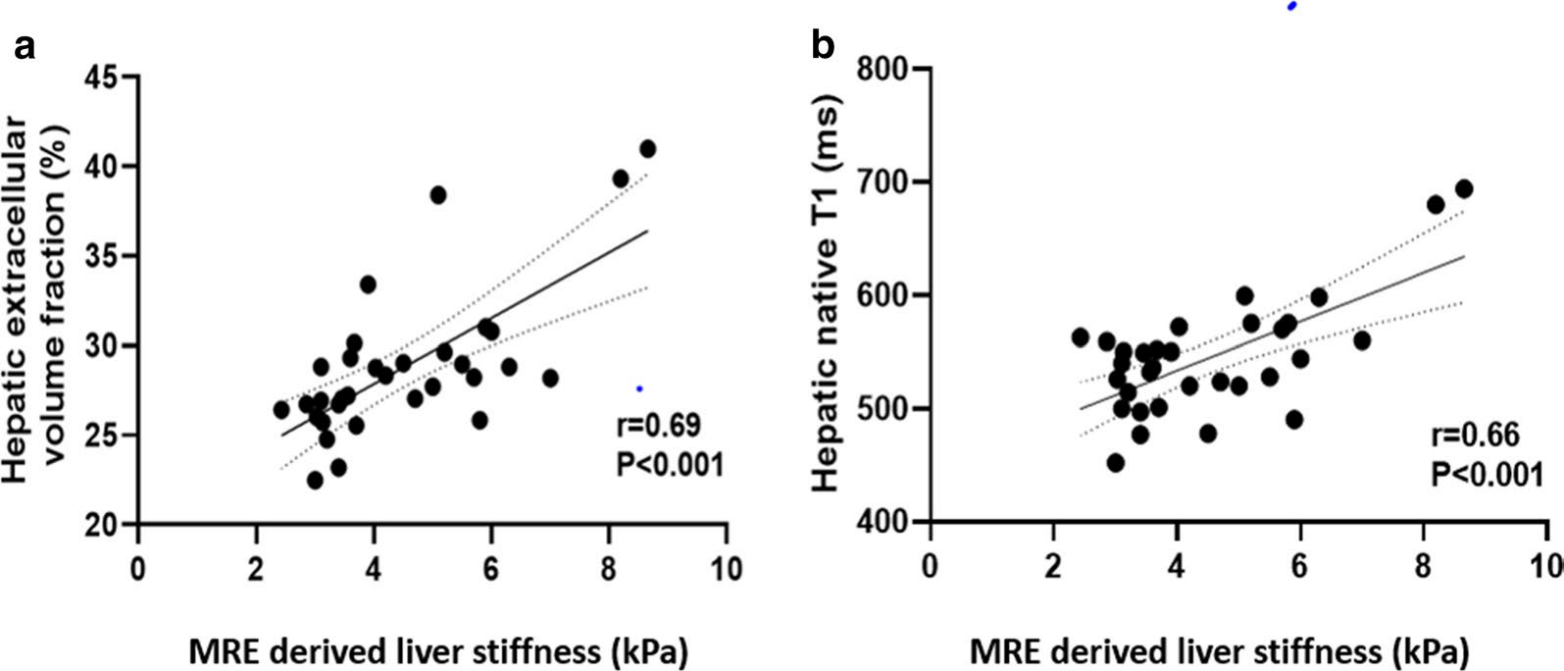
Scatter plots shows correlations between magnetic resonance elastography (MRE) derived liver stiffness and hepatic extracellular volume fraction (**a**) and hepatic native T1 (**b**). Regression lines are given with 95% confidence intervals

According to both, the AASLD and EASL guidelines [5, 6, 33], imaging plays a fundamental role in the management of PSC patients, since it is essential for confirming the diagnosis of PSC in the majority of patients and aids in assessment of disease progression and identification of possible complications and associated diseases, especially cholangiocarcinoma. MRI as a modality of choice for liver parenchyma characterization may possibly replace both invasive procedures and non-specific clinical scores. Another modality, which has proven to be effective in detecting significant fibrosis in patients with PSC is TE. Liver stiffness measurements derived by TE also showed correlations with hepatic ECV in our study. Considering the fact that MRE has proved to be a more accurate method for liver fibrosis assessment in patients with chronic liver disease compared to TE and the fact that TE was not performed in all patients at the time of MRI examination, we chose MRE-derived liver stiffness measurements as the main reference standard in our study [34]. In a previous study including 38 patients with PSC, the authors demonstrated high sensitivity and specificity of apparent diffusion coefficient (ADC) values in the detection of early (75% and 75%, respectively) and advanced (80% and 85%, respectively) liver fibrosis [35]. However, the usefulness of diffusion-weighted imaging for assessment and staging of liver fibrosis is still controversial due to existing limitations in MRI protocols and also standardization and ADC value reproducibility [33, 36]. Another promising MRI technique is relaxometry including ECV calculation. Significant correlations between hepatic T1, T2 as well as ECV with liver fibrosis have been already sufficiently described in the previous studies [17, 20, 21, 37–39]. Liver fibrosis is defined as the accumulation of extracellular matrix proteins produced by fibrogenic cell populations in response to tissue injury. As a consequence, this process leads to extension of extracellular space and increased accumulation of extracellular MRI contrast agent, which is reflected by prolonged T1 relaxation times and increased ECV of liver [21]. However, there is still no sufficient data proving correlations between MRE-derived liver stiffness and mapping parameters in patients with PSC. Using MRE-derived liver stiffness as a reference standard, we found strong correlations between hepatic ECV and liver stiffness (r = 0.69, *P* < 0.001) in patients with PSC (see also Fig. 3). Moreover, hepatic ECV showed a high diagnostic performance to diagnose significant fibrosis (F ≥ 2) in patients with PSC (AUC of 0.858). The diagnostic performance of ECV was higher than that of all non-invasive laboratory tests under investigation. One of the most important drawbacks of all laboratory tests and clinical scores is that they are not liver-specific. As a result, fibrotic and inflammatory changes outside of the liver contribute to bias. This is of particular importance in the PSC group where the prevalence of comorbidities is commonly high. In particular, in cases with accompanying diseases, which are typical in patients with PSC. In contrast, quantitative mapping parameters reflect the changes in the liver parenchyma itself. Moreover, one of the main advantages of ECV calculation is that compared to conventional T1 and T2 mapping, ECV is relatively independent of field strength and acquisition parameters, and, thus, can be considered as a physiologically normalized measure.

Native hepatic T1 also demonstrated high diagnostic performance in diagnosing significant fibrosis (r = 0.66, *P* < 0.001, see also Fig. 3). For the same reasons as ECV, T1 mapping is more liver specific and reflects the changes in liver parenchyma itself. However, in contrast to ECV, T1 parameters are less sensitive (84.2% vs. 52.6%), which could be explained by the dependency on technical aspects, as mentioned previously, and by heterogeneous nature of hepatic fibrosis in patients with PSC.

Our study has several limitations. The main drawback of our study is the absence of liver biopsy as a “gold standard”. Liver biopsy is an invasive procedure, which carries risks of periprocedural complications, and is also limited by sampling error due to disease heterogeneity in PSC. Thus, liver biopsy is no longer performed routinely for PSC and cannot be employed as a reference standard for PSC studies. Thus, we used MRE-derived liver stiffness as a reference standard for liver fibrosis assessment in our study. Another limitation is that we did not obtain full coverage of the liver parenchyma. Only a single transverse section for acquisition of T1 and T2 maps at the level of portal vein bifurcation was performed, which may have missed other significant changes, potentially occurring in other planes. As we additionally excluded the patients with iron overload and/or steatosis, our T1 measurements were not corrected for that. Moreover, similar to MRE, T1 and T2 mapping overestimate the degree of liver fibrosis in patients with inflammation or vascular congestion, highlighting these other factors that might affect T1 and T2 relaxation times. Additionally, the small sample size and the fact that most patients in our study cohort had fibrosis stage F4 might also limit the applicability of our results. Further studies with large patient cohorts using a liver biopsy as the main reference standard as well as other serological fibrosis scores (e.g. enhanced liver fibrosis test) are needed to establish the results of this study and confirm the accuracy and usefulness of MRI mapping in patients with chronic liver disease.

## Conclusions

In our observational prospective study, MRI mapping parameters, including ECV calculation showed strong correlations with MRE-derived liver stiffness. Especially, T1 mapping techniques with estimation of ECV have a potential to be a new non-invasive biomarker for assessment and early detection of significant fibrosis in patients with PSC by providing additional information, without adding costs to examination.

## Data Availability

The datasets generated and/or analysed during the current study are not publicly available due to privacy and ethical restriction but are available from the corresponding author on reasonable request.

## Abbreviations

PSC: Primary sclerosing cholangitis
MRI: Magnetic resonance imaging
MRE: Magnetic resonance elastography
ECV: Extracellular volume fraction
AUC: Area under the curve
